# Serum prolactin levels and sexual dysfunction in patients with schizophrenia treated with antipsychotics: comparison between aripiprazole and other atypical antipsychotics

**DOI:** 10.1186/s12991-017-0166-y

**Published:** 2017-11-28

**Authors:** Eiji Kirino

**Affiliations:** 1 Department of Psychiatry, Juntendo University Shizuoka Hospital, 1129 Nagaoka, Izunokunishi, Shizuoka 4102211 Japan; 2 Department of Psychiatry, Juntendo University School of Medicine, 2-1-1 Hongo, Bunkyoku, Tokyo 1138421 Japan; 3 Juntendo Institute of Mental Health, 700-1 Fukuroyama, Koshigayashi, Saitama 3430032 Japan

**Keywords:** Schizophrenia, Hyperprolactinemia, Antipsychotics, Aripiprazole, Sexual dysfunction

## Abstract

**Objectives:**

Antipsychotics, even atypical ones, can induce hyperprolactinemia. Aripiprazole (APZ), a dopamine D2 partial agonist, has a unique pharmacological profile and few side effects. We investigated the incidence of hyperprolactinemia in patients with schizophrenia treated with APZ and other antipsychotics.

**Methods:**

Serum prolactin levels were measured by ELISA (enzyme-linked immunosorbent assay). A questionnaire survey was used to evaluate subjective sexual dysfunction.

**Results:**

Based on the results of the questionnaire, approximately half (48.1%) of the patients complained of sexual dysfunction. The serum prolactin levels were significantly higher in patients with sexual dysfunction than in those without. In patients treated with antipsychotic monotherapy, the serum prolactin levels were significantly lower in patients treated with APZ than with other antipsychotics. In patients receiving 2 or more antipsychotics, the serum prolactin levels were significantly lower in patients treated with APZ-containing regimens than in patients treated with APZ-free regimens.

**Conclusions:**

Treatment with APZ did not influence the serum prolactin level, and adjunctive treatment with APZ may ameliorate the hyperprolactinemia that occurs during monotherapy with other antipsychotics.

## Introduction

Antipsychotics are an effective pharmacological therapy for schizophrenia. Second-generation antipsychotics (SGAs) have been developed recently that have effects on positive symptoms that are similar to those of first-generation antipsychotics (FGAs) plus a lower incidence of adverse reactions, such as extrapyramidal symptoms (EPSs). Although SGAs are not a homogeneous group [1–4], some SGAs have more promising effects on negative symptoms than FGAs [5, 6]. However, even SGAs that have been demonstrated to be safe have been found to induce weight gain, disturb glucose/lipid metabolism, and cause hyperprolactinemia [7]. Hyperprolactinemia that is related to excessive blockage of funnel/pituitary gland system D2 receptors is closely associated with sexual dysfunction, including irregular menstruation and erectile insufficiency, decreases in bone density after long-term use, and the risk of breast cancer [8–10]. However, it can be difficult to detect side effects such as sexual dysfunction that may contribute to recurrent schizophrenia due to self-discontinuation of drug therapy. Although FGAs were initially thought to be responsible for antipsychotic-induced hyperprolactinemia, SGAs have also been implicated. Leucht et al. [11] in a meta-analysis comparing the tolerability of 15 antipsychotics reported that most SGAs elevated serum prolactin levels significantly.

Aripiprazole (APZ) has a unique pharmacological profile and is a partial agonist of dopamine D2 and serotonin 5-HT1A receptors and an antagonist of the serotonin 5-HT2A receptor. This drug has few of the typical side effects of other antipsychotic drugs, such as EPSs, hyperprolactinemia, weight gain, metabolic disorders, and sedation [12]. APZ appropriates intrinsic activity at dopamine D2 receptors, thereby stabilizing dopamine D2 receptor-mediated neurotransmission without excessive blocking. Because of this, APZ is often called a dopamine system stabilizer [13].

Kapur et al. [14] proposed that antipsychotics with fast dissociation from the D2 receptor are more accommodating of physiological dopamine transmission, achieving an antipsychotic effect without motor side effects, prolactin elevation, or secondary negative symptoms. That group argued that the atypical antipsychotic effect, which they called “atypicality,” can be produced by appropriate modulation of the D2 receptor alone and that the blockade of other receptors is neither necessary nor sufficient.

In line with these findings, Kane et al. [15] reported a multicenter, randomized, double-blind, placebo-controlled 16-week study of adjunctive APZ for patients with schizophrenia or schizoaffective disorder treated with quetiapine (QTP) or risperidone (RIS) monotherapy. Adjunctive APZ was associated with significantly greater decreases in mean serum prolactin levels compared to baseline versus the adjunctive placebo (− 12.6 ng/mL for APZ vs − 2.2 ng/mL for placebo; *P* < 0.001). This effect was seen in the RIS subgroup (− 18.7 ng/mL vs − 1.9 ng/mL; *P* < 0.001) but not in the QTP subgroup (− 3.01 ng/mL vs + 0.15 ng/mL; *P* = 0.104). Similar reductions in prolactin levels were seen following adjunctive use of APZ in haloperidol (HPD) treated patients with hyperprolactinemia [16].

The present study investigated the incidence of hyperprolactinemia in patients with schizophrenia treated with APZ and other antipsychotics. In addition, we conducted a survey about sexual dysfunction using an original questionnaire.

## Subjects and methods

This non-blinded open study evaluated the serum prolactin level and subjective sexual dysfunction as well as the positive and negative syndrome scale (PANSS) score and the Clinical Global Impressions-Severity (CGI-S) score. The subjects were outpatients with schizophrenia (ICD-10, DSM-4-TR/DSM-5: schizophrenia, schizoaffective disorder, or schizotypal disorder, or schizoid personality) who were aged 17–60 years who were treated at the Department of Psychiatry at the Juntendo University Shizuoka Hospital between June 1, 2010 and May 31, 2017 and who received 1 or more antipsychotic medication. The subjects were serially registered during the entry period.

**Fig. 1:**
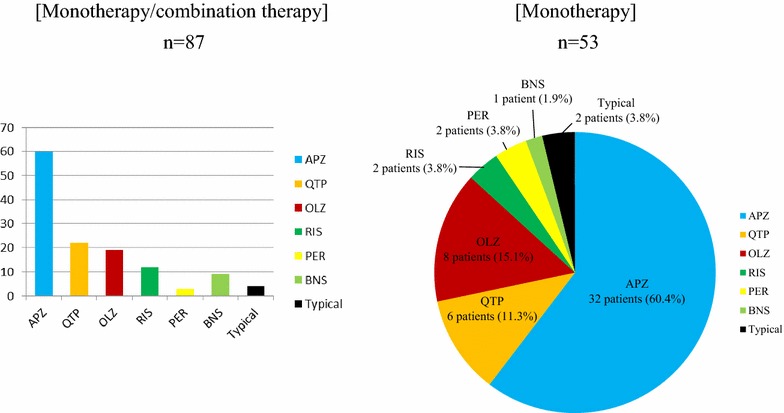
Antipsychotics prescribed in the sample population. APZ was most frequently prescribed in patients treated with monotherapy or combination therapy. *APZ* aripiprazole, *BNS* blonanserin, *OLZ* olanzapine, *PER* perospirone, *RIS* risperidone, *QTP* quetiapine

There were 87 subjects, including 36 males (41.4%) and 51 females (58.6%). The mean age was 39.16 ± 14.11 years (males: 38.81 ± 13.72; females: 39.41 ± 14.51). The patient age distribution was as follows: 10–19 years, 11 (12.6%); 20–29 years, 13 (14.9%); 30–39 years, 21 (24.1%); 40–49 years, 25 (28.7%); and others, 17 (19.5%). Of the 87 subjects, 67 (77.0%) were diagnosed with schizophrenia, 13 (14.9%) with schizoaffective disorder, and 7 (8.1%) with other disorders (schizotypal disorder, schizoid personality). A total of three patients (3.4%) had acute conditions, and 83 (95.4%) had chronic conditions. The mean duration of illness was 12.40 ± 10.58 years, and 19 patients (21.8%) had a history of hospital admission. Complications were noted in eight patients (Table 1). While 85 patients (97.7%) had received atypical agents, four had received (4.6%) typical agents (including combination therapy). The atypical agents included APZ in 60 patients (69.0%), QTP in 33 (37.3%), olanzapine (OLZ) in 19 (21.8%), and RIS in 12 (13.8%). Antipsychotic monotherapy had been prescribed to 53 patients (60.9%); of these, 32 (60.4%) had received APZ, the largest population for any agent (Fig. 1).

**Table 1:** Demographic and clinical characteristics of the patient sample

Number of patients	87
Gender	Male: 36 (41.4%), Female: 51 (58.6%)
Age (mean ± SD)	39.16 ± 14.11 years Male: 38.81 ± 13.72 years, Female: 39.41 ± 14.51 years
Diagnosis	Schizophrenia: 67 (77.0%) Schizoaffective disorder: 13 (14.9%) Others: 7 (8.1%)
Acute/chronic phases	Acute phase: 3 (3.4%), chronic phase: 83 (95.4%), unclear: 1 (1.1%)
Duration of illness (mean ± SD)	12.40 ± 10.58 years
History of admission	Present: 19 (21.8%), absent: 67 (77.0%), unclear: 1 (1.1%) Previous admission, once: 11, twice: 5, 3 times: 2, 4 times: 1
Complication	Present: 8 patients (9.2%) Autoimmune hepatitis in 1 patient, lung cancer in 1, drug-induced hepatopathy in 1, breast cancer in 1, uterine cancer/A–V block in 1, sarcoidosis in 1, anomaly of the cerebral artery in 1, and congestive heart failure in 1

An ELISA kit (enzyme-linked immunosorbent assay) (Bioclone Australia Pty Limited, Marrickville NSW Australia) was used to measure serum prolactin levels. We prepared an original questionnaire survey about subjective sexual dysfunction to investigate whether each subject experienced erectile insufficiency, projectile ejaculation/semen, irregular menstruation, breast tension/phyma, breast milk production, or decreased sexual libido.

For statistical analysis, alpha values of 0.05 were considered significant in paired *t* tests and for Spearman’s rank correlation coefficients.

## Results

**Fig. 2:**
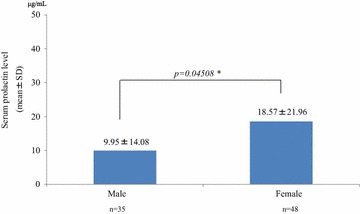
Gender differences in serum prolactin levels. The mean serum prolactin level in females was significantly higher than the mean level in males

The mean serum prolactin level in females (18.57 ± 21.96 μg/mL) was significantly higher than in males (9.95 ± 14.08 μg/mL) (*P* = 0.04508). The normal range is 1.5–9.7 μg/mL in males and 1.4–14.6 μg/mL in females (Fig. 2). There were 10 males (27.8%) and 16 females (31.4%) with abnormal values, and some had a serum prolactin level > 100 μg/mL (1 female during OLZ 20 mg/day monotherapy and 1 female during RIS 3 mg/day monotherapy). The latter two patients reported irregular or no menstruation on the survey sheet. There were no significant correlations between serum prolactin level and age, the number of antipsychotics, the dose of the antipsychotics (chlorpromazine equivalent), or the duration of illness when calculating Spearman’s rank correlation coefficient.

**Fig. 3:**
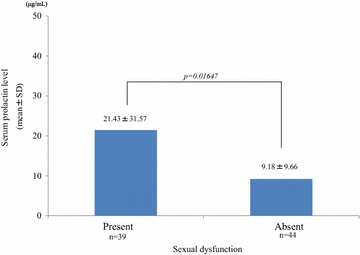
The contribution of the presence of sexual dysfunction to serum prolactin levels. The serum prolactin levels were significantly higher in patients who checked at least 1 sexual dysfunction item on the questionnaire versus those who did not

**Fig. 4:**
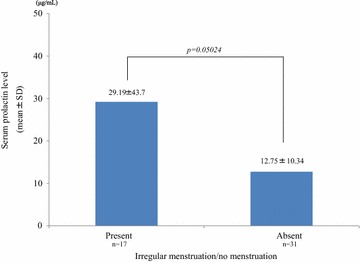
The contribution of the presence of menstruation abnormalities to serum prolactin levels. Although not statistically significant, the serum prolactin levels in females who reported irregular menstruation tended to be higher than in those who did not

Of the 81 patients (collection rate: 93.1%) from whom we collected survey sheets, 39 (48.1%) checked items indicating sexual dysfunction. In this sexual dysfunction group, the mean serum prolactin level (21.43 ± 31.57 μg/mL) was significantly higher than in subjects who did not check any items (non-sexual dysfunction group; 9.18 ± 9.66 μg/mL) (*P* = 0.01647) (Fig. 3). Although this was not statistically significant, the serum prolactin levels in females complaining of irregular menstruation tended to be higher than the levels in those without this problem (29.19 ± 43.7 vs 12.75 ± 10.34 μg/mL; *P* = 0.05024) (Fig. 4).

**Fig. 5:**
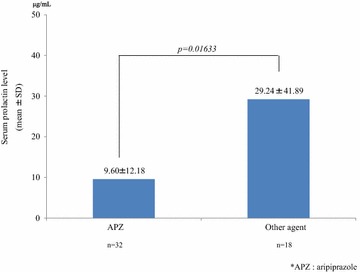
The effect of monotherapy with antipsychotics on the serum prolactin level: APZ versus other agents. In patients treated with monotherapy, the serum prolactin levels in patients treated with APZ were significantly lower than in those treated with other antipsychotics

**Fig. 6:**
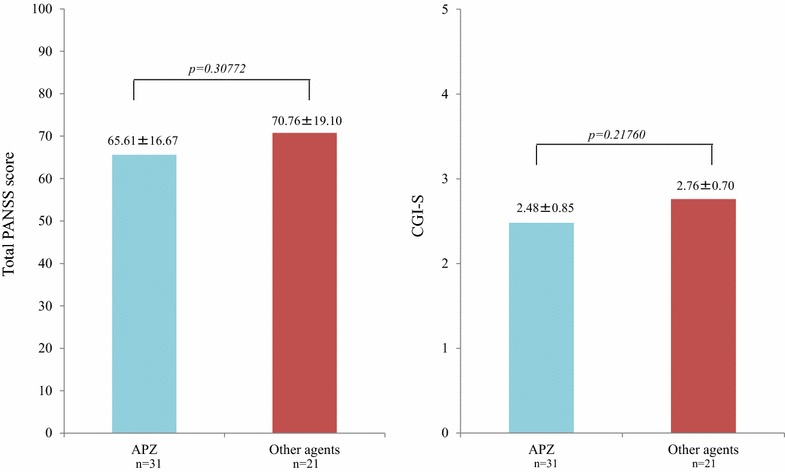
The effect of monotherapy with antipsychotics on the total PANSS score and the CGI-S score: APZ versus other agents. There were no significant differences in the mean total PANSS score or CGI-S score between the APZ group versus other antipsychotic groups

In patients receiving monotherapy, the mean serum prolactin level in patients treated with APZ was significantly lower than in those treated with other antipsychotics (9.60 ± 12.18 μg/mL vs 29.24 ± 41.89 μg/mL; *P* = 0.01633) (Fig. 5). There were no significant differences in the total mean PANSS scores in the APZ group vs the other antipsychotic group (65.61 ± 16.67 vs 70.76 ± 19.10; *P* = 0.30772). Similarly, there were no significant differences in the mean CGI-S score in the APZ group vs the other antipsychotic group (2.48 ± 0.85 vs 2.76 ± 0.70; *P* = 0.21760) (Fig. 6).

**Fig. 7:**
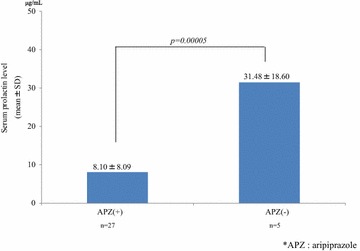
The effect of APZ-containing regimens versus APZ-free regimens in combination therapies on the serum prolactin level. In patients receiving 2 or more antipsychotics, the serum prolactin levels in patients treated with APZ-containing regimens were significantly lower than the levels in those treated with APZ-free regimens

Regarding patients who were receiving 2 or more antipsychotics, the mean serum prolactin level in patients treated with APZ-containing regimens was significantly lower than in those treated with APZ-free regimens (8.10 ± 8.09 μg/mL vs 31.48 ± 18.60 μg/mL; *P* = 0.00005) (Fig. 7).

## Discussion

This study found that the serum prolactin level increased in some patients with schizophrenia who were treated with antipsychotics. Approximately half of the patients who completed the study questionnaire reported sexual dysfunction. Antipsychotic-associated sexual dysfunction can reduce patient compliance; accordingly, it may be important to evaluate the presence of sexual dysfunction in the early phase of treatment by communicating with the patient. Notably, the serum prolactin level may have increased in patients complaining of sexual dysfunction.

Baggaley [17] reported that the incidence of sexual dysfunction ranged from 30 to 80% in patients with schizophrenia who were not treated or who were receiving treatment. The incidence was higher than in patients with other psychiatric disorders, suggesting that sexual dysfunction reduces the patient’s quality of life. Fujii et al. [18] indicated that clinicians must consider the risk of sexual dysfunction because the incidence was higher in patients with schizophrenia than in healthy adults in Asia. The Expert Consensus Guideline 2009 [19] emphasized that sexual dysfunction is an important side effect of antipsychotics that is associated with compliance.

In the present study, the serum prolactin level in females was significantly higher than in males. Kleinberg et al. [20] measured the serum prolactin level in patients with schizophrenia during RIS or HPD therapy and also found that the serum prolactin level was higher in females than in males, although the values for both sexes were higher than in the placebo group, regardless of dose. Jerrell et al. [21] reported that in patients receiving antipsychotics, the incidence of sexual dysfunction was higher in females, adolescent patients, patients receiving an SSRI or valproate, obese patients, and those with endocrine disturbances. Therefore, factors other than the type of antipsychotic, including gender, age, and concurrent agents, should be considered when treating schizophrenia as these factors may cause sexual dysfunction.

In this study, 48.1% of the patients indicated that at least 1 sexual dysfunction item was present during antipsychotic therapy. However, Cutler [22] reported that the incidence of sexual dysfunction that was predicted by the psychiatrists of patients with schizophrenia during antipsychotic therapy was 10–20% lower than the actual incidence of sexual dysfunction that patients subjectively experienced. In clinical practice, it may be necessary to investigate the presence of sexual dysfunction in patients treated with antipsychotics by asking them directly or by using a questionnaire. The serum prolactin level was significantly higher in patients who reported at least 1 sexual dysfunction item than in those who did not; therefore, the serum prolactin level should be measured in patients who complain of sexual dysfunction. Citrome [23] argued that there should be regular follow-up of the serum prolactin level after the start of antipsychotic therapy based on the patient’s medical history and manifestation of symptoms. This is in accordance with at least 7 guidelines for the administration of any antipsychotic that may cause sexual dysfunction, i.e., the guidelines of the American Psychiatric Association; Mount Sinai Conference; Expert Consensus Survey; Canadian Psychiatric Association, Australia and New Zealand; UK National Institute for Health and Clinical Excellence 2006; and the Maudsley Prescribing Guidelines. Therefore, it is recommended that the baseline serum prolactin level should be measured initially and then monitored regularly [23].

The present study found that the serum prolactin level increased in the sexual dysfunction group. Furthermore, some of the patients in this study showed abnormally high prolactin levels that exceeded 100 μg/mL, and these patients reported irregular or no menstruation on the questionnaire. In the other patients, the serum prolactin levels in females who reported irregular menstruation tended to be higher than in those without this problem. However, there was no correlation between the number or dose of antipsychotics, duration of illness, or prolactin level, suggesting that follow-up of hyperprolactinemia or sexual dysfunction is necessary in both patients with long-term schizophrenia who receive massive doses of therapy with several antipsychotics and in patients with short-term schizophrenia who are receiving low-dose monotherapy.

Bostwick et al. [24] indicated that treatment should be switched to combination therapy with agents that do not influence the prolactin level, such as dopamine agonists, when there is an antipsychotic-related increase in the prolactin level, although this may compromise antipsychotic efficacy. In addition, the doses of agents that cause an elevation in prolactin should be decreased in patients that show a high serum prolactin level. Otherwise, agents that decrease the prolactin level, such as cabergoline and bromocriptine, should be added, or the patient should be treated with an agent such as APZ that does not influence the serum prolactin level, as reported by this study as well as others [15, 16, 25, 26]. Both strategies of switching to APZ [27–29] and addition of APZ [15, 30–32] to previous antipsychotics have been reported to be effective in resolving antipsychotic-induced hyperprolactinemia and hyperprolactinemia-related adverse events [33]. However, APZ being a partial agonist has a lower intrinsic activity at the D2 receptor than dopamine, allowing it to act as both, a functional agonist and antagonist, depending on the surrounding levels of dopamine [34]. Hence, it should be reminded in clinical scenes that APZ could act as a functional antagonist and thus elevate prolactin levels in the absence of a competing D2 antagonist and the presence of dopamine (the natural agonist) in some cases [34, 35].

We found a significant difference in the prolactin levels in patient groups treated with APZ-containing regimens vs APZ-free regimens, which is in line with previous findings [15, 16]. Adjunctive APZ with RIS may optimize D2 receptor activity and, hence, diminish the risk for EPS associated with RIS [36] as well as decrease prolactin elevation resulting from high D2 receptor occupancy by a full antagonist [16]. Thus, adjunctive APZ may potentially ameliorate the side effects that occur during monotherapy with other antipsychotics [15].

This study had some limitations. Our present findings are underpowered due to our relatively small sample. Furthermore, the sampling was biased in that the number of patients treated with APZ was much higher than the number of those treated with other antipsychotics. We should be prudent in interpreting our results although some of the analyses were statistically significant. Elucidating the clinical implications of our present findings will require further randomized or blinded studies in a larger sample.

## Conclusions

Treatment with APZ did not influence the serum prolactin level, and adjunctive treatment with APZ may ameliorate the hyperprolactinemia that occurs during monotherapy with other antipsychotics.
